# Connexin43 is Dispensable for Early Stage Human Mesenchymal Stem Cell Adipogenic Differentiation But is Protective against Cell Senescence

**DOI:** 10.3390/biom9090474

**Published:** 2019-09-11

**Authors:** Qing Shao, Jessica L. Esseltine, Tao Huang, Nicole Novielli-Kuntz, Jamie E. Ching, Jacinda Sampson, Dale W. Laird

**Affiliations:** 1 Department of Anatomy and Cell Biology, Schulich School of Medicine and Dentistry, University of Western Ontario, London, ON N6A 5C1, Canada; cindy.shao@schulich.uwo.ca (Q.S.); huangtaorrr@163.com (T.H.); nicole.kuntz@uwo.ca (N.N.-K.); 2 Division of BioMedical Sciences, Memorial University of Newfoundland, St. John’s, NL A1B 3V6, Canada; jesseltine@med.mun.ca; 3 Department of Pathology, Shenyang Medical College, Shenyang 110034, China; 4 Department of Physiology and Pharmacology, Schulich School of Medicine and Dentistry, University of Western Ontario, London, ON N6A 5C1, Canada; jching8@uwo.ca; 5 Department of Neurology, Stanford University Medical Center, Palo Alto, CA 94304, USA; jacindas@stanford.edu

**Keywords:** mesenchymal stem cells, adipogenesis, connexin43, gap junctional intercellular communication, oculodentodigital dysplasia, senescence, CRISPR-Cas9

## Abstract

In the last couple of decades, there has been a growing optimism surrounding the potential transformative use of human mesenchymal stem cells (MSCs) and human-induced pluripotent stem cells (iPSCs) for regenerative medicine and disease treatment. In order for this to occur, it is first essential to understand the mechanisms underpinning their cell-fate specification, which includes cell signaling via gap junctional intercellular communication. Here, we investigated the role of the prototypical gap junction protein, connexin43 (Cx43), in governing the differentiation of iPSCs into MSCs and MSC differentiation along the adipogenic lineage. We found that control iPSCs, as well as iPSCs derived from oculodentodigital dysplasia patient fibroblasts harboring a *GJA1* (Cx43) gene mutation, successfully and efficiently differentiated into LipidTox and perilipin-positive cells, indicating cell differentiation along the adipogenic lineage. Furthermore, the complete CRISPR-Cas9 ablation of Cx43 from iPSCs did not prevent their differentiation into bona fide MSCs or pre-adipocytes, strongly suggesting that even though Cx43 expression is upregulated during adipogenesis, it is expendable. Interestingly, late passage Cx43-ablated MSCs senesced more quickly than control cells, resulting in failure to properly differentiate in vitro. We conclude that despite being upregulated during adipogenesis, Cx43 plays no detectable role in the early stages of human iPSC-derived MSC adipogenic differentiation. However, Cx43 may play a more impactful role in protecting MSCs from premature senescence.

## 1. Introduction

The use of human stem cells in regenerative medicine has become an intriguing area of research owing to the fact that stem cells with great differentiation capacity can be obtained from a number of different sources [1,2]. Mesenchymal stem cells (MSCs) are multipotent cells capable of differentiating into osteoblasts, chondrocytes, adipocytes, and other fully differentiated cell types [3,4]. MSCs have extensive utility as they are subject to paracrine signaling, immunomodulation, differentiation, and trans-differentiation, all of which support their potential use in the clinic [1,2,3,4,5,6]. As of 2016, there were nearly 500 clinical trials centered on the use of MSCs for the treatment of a variety of human pathologies including cardiac infarction, bone and joint disease, and neurodegeneration [5]. MSCs can be isolated from a number of adult human tissues including bone marrow, adipose tissue, dental pulp, and umbilical cord blood, generating a heterogeneous and finite supply of cells directly from a patient source [7]. With the advent of human-induced pluripotent stem cells (iPSCs) derived from fully differentiated cells, which can readily be differentiated into MSCs, there arose a potentially infinite source of pure MSCs for research and potential therapeutic use. However, there is critical need to understand the factors that regulate their self-renewal and differentiation, as well as their tolerance and robustness to genetic modification and cell passage. Here, we addressed how connexin43 (Cx43) may participate and regulate iPSC and MSC differentiation along the adipogenic lineage in culture.

Gap junctional intercellular communication (GJIC) plays a role in coordinating the complex series of events necessary to maintain progenitor and stem cell survival, and for cells to execute terminal differentiation [8,9,10]. GJIC facilitates direct signaling between neighboring cells via the cytoplasmic exchange of small molecules through docked connexin hemichannels found between adjacent cells [11]. Undocked connexin hemichannels have also been shown to play some role in mediating autocrine and paracrine signaling, although their role beyond pathological cell processes remains poorly understood in vivo [12]. MSCs, adipocyte-derived stromal cells, and bone marrow-derived stromal cells all express the prototypical connexin, Cx43 [13,14,15,16,17,18]. Cx43 is widely found in the human anatomy, and more than 80 different mutations in the gene encoding Cx43 (*GJA1*) are associated with the pleiotropic developmental disorder known as oculodentodigital dysplasia (ODDD). ODDD is commonly characterized by craniofacial malformations, enamel hypoplasia, syndactyly, ocular deficits, and other less penetrant organ anomalies [10,19,20,21,22,23]. Many unaffected organs and tissues also express Cx43 but appear to be protected from anomalies, possibly by the co-expression of other connexin family members. However, this does not appear to be the case in fat, as adipocytes only express Cx43 and there is no evidence in ODDD patients that white or brown fat formation is abnormal. Yet, Cx43 has recently been proposed to play a role in adipose-derived stromal cell differentiation into adipocytes, as well as adipose beiging, in rodents and humans [15,24,25,26,27,28].

Another consequence of dysregulating Cx43 expression in stem or progenitor cells is the possibility that this defect could drive putatively differentiating cells into senescence. For example, Cx43 downregulation has been shown to accelerate the senescence of human fibroblasts [29], rat aortic endothelial cells [30], and mouse hematopoietic stem cells [31]. Several age-related conditions are attributed to stem cell depletion through senescence of skeletal muscle stem cells, neural stem cells, and others [32,33]. The role of GJIC in stem cell senescence is not fully understood, but cell communication is central to this process as senescent cells possess a unique senescence-associated secretory phenotype [34]. Cellular senescence is also frequently associated with DNA damage, oxidative stress, mitochondrial dysfunction, and niche degeneration which is mediated through retinoblastoma activity, as well as p53 and the master regulator of senescence, p16INK4a [35].

Here, we assessed the role of Cx43 in adipogenic differentiation from MSCs where Cx43 was either rendered functionally compromised by harboring an ODDD-linked *GJA1* gene mutation or ablated. We also examined how Cx43 ablation or dysfunction impacts the differentiation capacity and onset of senescence in late-passage stem cells.

## 2. Materials and Methods

### 2.1. Human iPSC Cultures

Previously described human iPSCs derived from dermal fibroblasts [10] (University of Western Ontario Research Ethics Board (104190), and the Institutional Review Board (00040092) from the University of Utah, in keeping with the Declaration of Helsinki principles) were cultured at 37 °C in humidified air with 5% CO_2_ under feeder-free conditions using Geltrex coating media (ThermoFisher #A1413302, Waltham, MA, USA) and Essential 8 (E8) stem cell media (ThermoFisher #A1517001) as described [10,36]. E8 media was replaced daily and iPSC colonies were monitored for spontaneous differentiation. For cell passaging, cells were incubated in enzyme-free Cell Dissociation Buffer (ThermoFisher #13151014) until colonies broke apart (~5 min) [37]. When the dissociation buffer was aspirated, cells were returned to E8 media, scraped into cell clumps, and re-seeded as small clumps onto Geltrex pre-coated dishes at 37 °C in humidified air with 5% CO_2_. Typically, cells were passaged approximately every seven days at a ratio of 1:6. All experiments were conducted using cells between passages 21–33.

### 2.2. MSC Differentiation and Culture

MSCs were differentiated from a healthy control relative and ODDD patient iPSCs (harboring a Cx43 p.V216L mutant) that were originally derived from dermal fibroblasts [10], or iPSCs where Cx43 was ablated (referred to here as Cx43-/- iPSCs), using the STEMdiff mesenchymal progenitor kit (StemCell Technologies #05240, Vancouver, BC) according to the manufacturer’s instructions. MSCs were cultured on gelatin-coated dishes in MesenCult-ACF basal media (StemCell Technologies #05445) in a 37 °C humidified incubator under 5% CO_2_. MSCs were passaged using the ACF-free cell dissociation kit (StemCell Technologies #05426). Cells at passages 3–5 were considered early passage, while cells at passages 9–12 were defined as late passage.

### 2.3. CRISPR-Cas9 Gene Ablation

iPSCs were transiently transfected using Lipofectamine 3000 (ThermoFisher #L3000015) with the pSpCas9(BB)-2A-GFP plasmid (PX458, Addgene, Watertown, MA, USA), which encodes for the Cas9 protein along with a cloning backbone for sgRNA [38]. Cells harboring a CRISPR-Cas9 targeted knockout of the gene encoding Cx43 were sorted and selected for Cx43 ablation. At least two Cx43 ablated cell clones were routinely used in subsequent experiments.

### 2.4. Flow Cytometry

Putative MSCs at passages 3–9 were analyzed via flow cytometry for the appropriate cell surface markers as the minimal experimental criteria for MSCs as per the International Society for Cellular Therapy: >95% positive for CD73-FITC (eBioscience clone AD2, ThermoFisher); >95% positive for CD105-PE (eBioscience clone SN6); <2% positive for CD34-eFluor450 (eBioscience clone 4H11); <2% positive for CD45-APC (eBioscience clone 2D1) [39]. Briefly, cells in suspension were incubated with the appropriate fluorescently conjugated primary antibody (1:500) for 45 min at room temperature. After three washes with PBS, cells were suspended in 4% paraformaldehyde and analyzed via flow cytometry (BD FACSCanto cytometer, San Jose, CA, USA). Fluorescence compensation and possible non-specific fluorescence were assessed using single-color and fluorescence minus one (FMO) controls for each color. Data were analyzed using FlowJo X pro software (Ashland, OR, USA).

### 2.5. Adipogenic Differentiation of MSCs 

Control, ODDD patient, and Cx43-/- human iPSC-derived MSCs were cultured on gelatin-treated dishes with glass cover slips in MesenCult-ACF medium (StemCell, Technologies, Vancouver, Canada). Once cells reached confluency, media was replaced with StemPro Adipogenesis Differentiation Kit (ThermoFisher #A1007001) per the manufacturer’s instructions. Media was changed every 2–3 days during the differentiation period of up to 28 days. At select intervals, cells were processed for immunocytochemistry and Western blotting.

### 2.6. Immunocytochemistry Labeling and LipidTox Green Neutral Lipid Stain Analysis

MSCs, as well as day 0, 7, 14, and 28 differentiated cells, were fixed with a 10% neutral formaldehyde solution followed by permeabilization with 0.1% Triton X-100. Samples were blocked with 5% BSA in PBS for 30 min at room temperature prior to labeling with antibodies to the following: Cx43 (rabbit, 1:1000; Sigma-Aldrich; #C6219 St. Louis, MO, USA), perilipin (rabbit mAB, 1:100, Cell Signaling Technology #9349 Danvers, MA, USA). After washing, primary antibodies were followed by goat anti-rabbit secondary antibodies conjugated to Alexa 555 (1:1000; ThermoFisher; #A21425,) or goat anti-mouse secondary antibodies conjugated to Alexa 488 (1:1000; ThermoFisher; #A11070), and nuclei were stained with Hoechst (1:1000, ThermoFisher #H3570). Lipid accumulation was detected via LipidTox Green stain according to the manufacturer’s instructions (ThermoFisher #H34475). Samples were imaged using a Zeiss LSM 800 confocal microscope equipped with a 63x/1.30 oil lens. Representative cell images reflect cell labeling performed at least three times on similar cell passages cultured on separate days.

### 2.7. Scrape Loading Dye Transfer

To assess the contribution of Cx43 to functional GJIC in MSCs, scrape loading dye transfer assays were performed in WT and Cx43-/- MSCs. MSCs were grown to confluence in 60 mm dishes and washed with Hanks balanced salt solution (HBSS). Confluent cell cultures were scratched using a scalpel to injure a row of cells, thus allowing dye entry into damaged cells. Cells were then incubated in gap junction–permeable Lucifer yellow (1 mg/mL; ThermoFisher) and gap junction–impermeable sulfodextran rhodamine B (0.5 mg/mL; ThermoFisher) dye solutions for 5 min at 37 °C. Samples were imaged using a Zeiss LSM 800 confocal microscope. Six fields of view (WT, *n* = 4 plated experiments; Cx43-/-, *n* = 5 plated experiments) were imaged per dish. Lucifer yellow dye passage distance was quantified from after the first row of damaged cells at three locations along each side of the scrape per field of view using ImageJ (NIH, Bethesda, MD, USA; https://imagej.nih.gov/ij/).

### 2.8. Western Blot Analysis

Undifferentiated and adipogenic differentiated MSC lysates were separated via SDS-PAGE, transferred to nitrocellulose membrane, and immunoblotted using antibodies to the following: Cx43 (rabbit, 1:5000; Sigma-Aldrich, #C6219) CDKN2A/p16INK4a (rabbit, 1:1000, Abcam, #Ab10834), and glyceraldehyde 3-phosphate dehydrogenase (GAPDH, 1:5000; Millipore, Etobicoke, Canada; #MAB374). Primary antibody labeling was detected using IRDye 800 (1:5000; Pottstown, USA; #611-132-122) or Alexa Fluor 680 (1:5000; ThermoFisher; #A21076) goat anti-rabbit and anti-mouse secondary antibodies and analyzed on the LiCor detection system. The relative quantities of the immunoblotted proteins were normalized to GAPDH using ImageJ (NIH, Bethesda, MD, USA; https://imagej.nih.gov/ij/). Quantified Western blots were performed at least three times while other representative Western blots were repeated at least once using separate sets of cells.

### 2.9. Senescence-Associated-β-Galactosidase Assay

Cellular senescence was assessed using the β-galactosidase staining kit (Cell Signaling Technologies #9806) following the manufacturer’s instruction. Briefly, MSC cells were seeded on 0.1% gelatin coated coverslips and cultured for 24 h. After washing with PBS, cells were fixed for 15 min and stained overnight for β-galactosidase activity using X-gal solution (pH 6.0) at 37 °C and counterstained with hematoxylin for cellular morphology. Images were acquired on a Leica DMIL LED histology microscope equipped with a 40× lens.

### 2.10. Statistics

All values are presented as mean ± SEM unless otherwise indicated. All results were analyzed using one-way ANOVA followed by Tukey post-hoc. All analyses were done using Graphpad Prism 7.0 statistical software. *, *p* < 0.05; **, *p* < 0.01; ***, *p* < 0.001.

## 3. Results

### 3.1. Differentiation of iPSCs into MSCs Occurs Independent of Cx43

**Figure 1:**
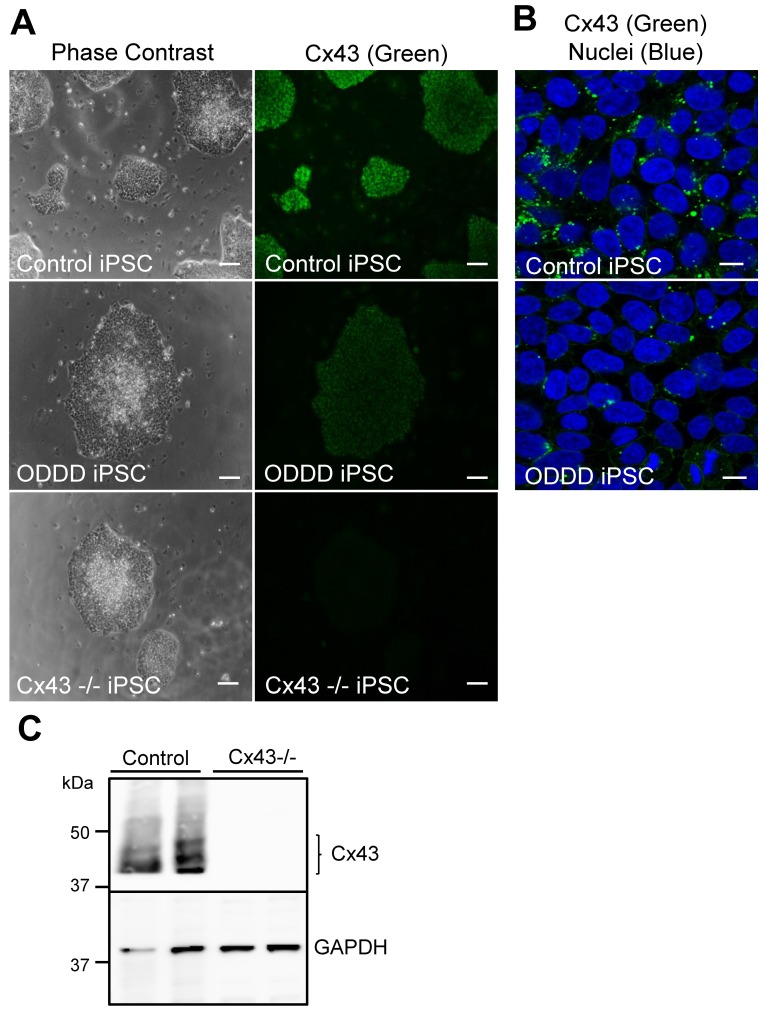
Connexin43 (Cx43) expression in control, oculodentodigital dysplasia (ODDD) patient-derived, and CRISPR-Cas9 knockout human iPSCs. (**A**) Human induced pluripotent stem cells (iPSCs) reprogrammed from control or ODDD patient-derived dermal fibroblasts, as well as CRISPR-Cas9-engineered Cx43-/- iPSCs, were immunofluorescently labeled for connexin43 (Cx43, green). Scale bar = 100 μm. (**B**) Confocal microscopy of Cx43 (green) in control and ODDD patient-derived iPSCs revealed the typical punctate distribution of Cx43 at cell-cell interfaces. Scale bar = 20 μm. (**C**) Representative immunoblots of Cx43 and glyceraldehyde 3-phosphate dehydrogenase (GAPDH) in two control, and two Cx43-/- iPSCs cell populations generated through CRISPR-Cas9 engineering.

**Figure 2:**
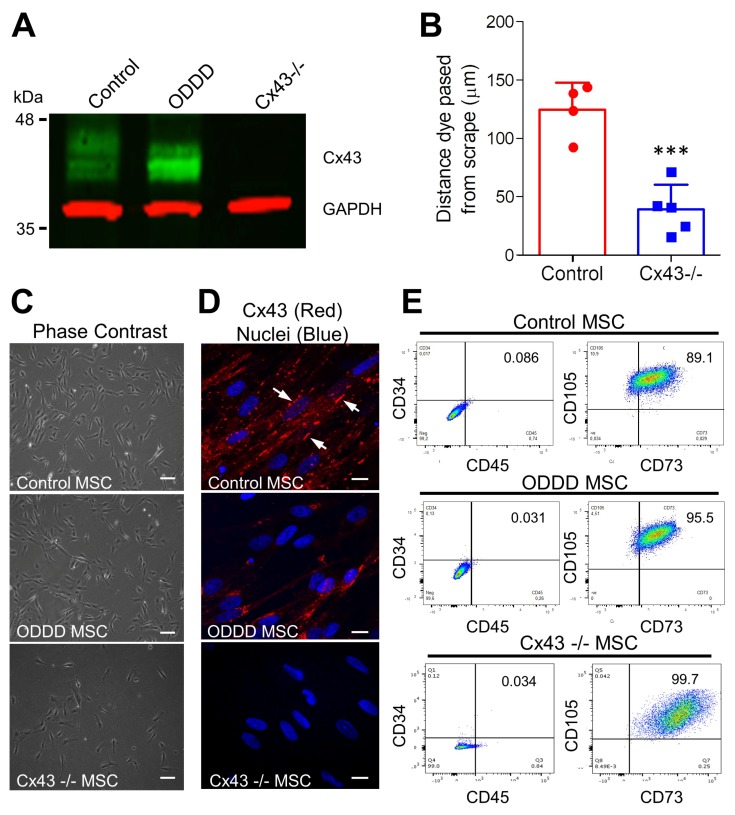
Mesenchymal stem cells (MSCs) established a prototypical cluster of differentiation (CD) expression profile expected of stem cells independent of Cx43 expression and functional status. (**A**) Representative immunoblot of Cx43 (green) and GAPDH (red) from control, ODDD patient-derived, and Cx43-/- iPSC-derived MSCs. (**B**) Scrape-loading transfer of gap junction channel-permeable Lucifer yellow revealed decreased GJIC in Cx43-/- MSCs compared to control cells. ***, *p* < 0.001 compared to WT. Data represent the standard deviation of the mean of 4–5 independent experiments comprising at least 144 individual measurements for each cell line. (**C**) Phase contrast and (**D**) immunofluorescent micrographs of Cx43 (red) and nuclei (Hoechst, blue) in control, ODDD patient-derived and Cx43-/- MSCs. Arrows indicate gap junction plaques between adjacent cells. Scale bar = 10 μm (**E**) MSCs differentiated from control, ODDD patient-derived and Cx43-/- iPSCs were analyzed by flow cytometry for the cell surface markers CD34, CD45, CD73, and CD105.

Control human iPSCs exhibited robust Cx43 expression (Figure 1), while ODDD patient-derived iPSCs, which harbor a genetic mutation (p.V216L), exhibited reduced Cx43 as previously shown (Figure 1A,B) [10]. Cx43-knockout iPSCs were generated via CRISPR-Cas9-mediated gene ablation and were found by immunofluorescence to be devoid of detectable Cx43 (Figure 1A,C). These results are consistent with a parallel study examining the dynamic regulation of connexins in stem cell pluripotency [40]. Independent of the Cx43 status, all three iPSC lines differentiated into adherent MSCs that typically grew as dispersed monolayers (Figure 2). Control MSCs expressed Cx43, which was localized to small punctate plaque-like regions at cell to cell interfaces (Figure 2A), while Cx43 was also expressed in ODDD patient-derived MSCs but was found more frequently within intracellular compartments (Figure 2D). Western blot confirmed Cx43 deletion in Cx43-/- MSCs, while scrape loading dye transfer of the gap junction permeable dye Lucifer yellow revealed reduced GJIC in Cx43-/- MSCs compared to control (Figure 2A,B). As per the International Society for Cellular Therapy guidelines, putative MSCs that contained high, low, and null levels of Cx43 exhibited stellate morphology and the ability to adhere to plastic (Figure 2C). The majority of cells expressed high levels of mesenchymal markers (CD73 and CD105), while very few cells expressed low levels of hematopoietic markers (CD34, CD45) (Figure 2E). Therefore, Cx43 mutation or genetic ablation does not negatively impact the ability of human iPSCs to differentiate in vitro into MSCs.

### 3.2. Cx43 is Upregulated during in vitro Adipogenic Differentiation of MSCs

**Figure 3:**
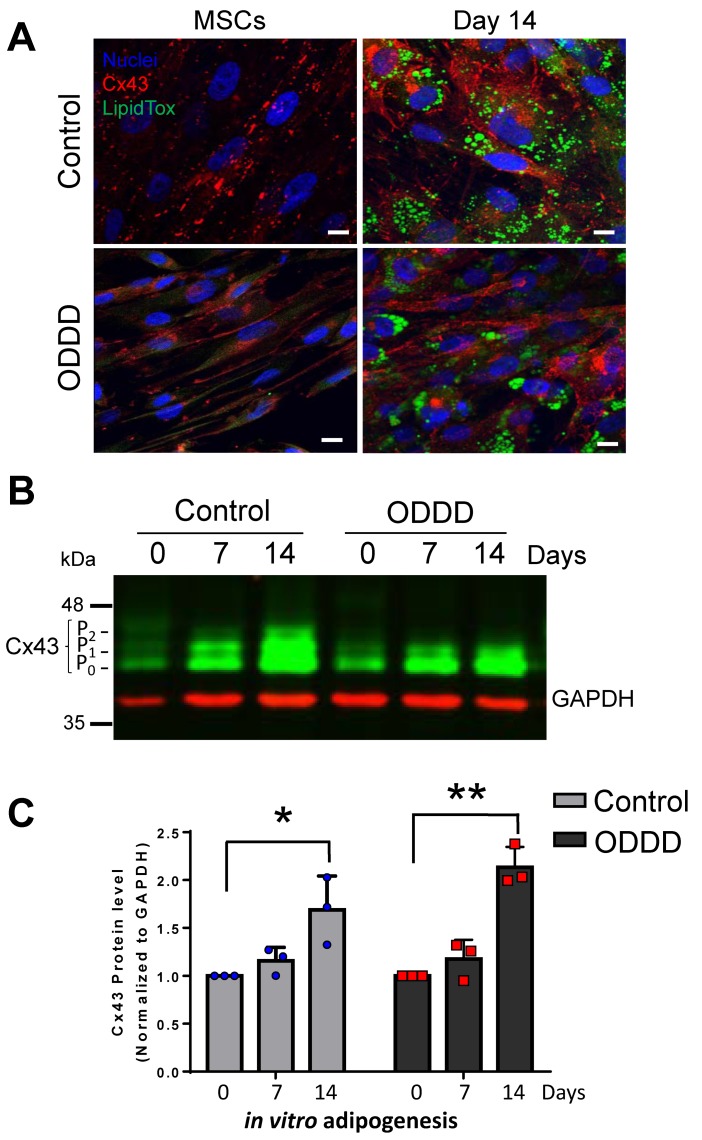
Cx43 was significantly upregulated during adipogenic differentiation of MSCs. (**A**) MSCs were cultured for up to 14 days under adipogenic conditions before labeling for Cx43 (red), lipid (green; LipidTox), and nuclei (Hoechst, blue). Scale bar = 10 μm. (**B**) Representative immunoblot of Cx43 (green) and GAPDH (red) in undifferentiated MSCs and MSCs differentiated along the adipogenic lineage for 7 and 14 days. The differential phosphorylation states of Cx43 are noted as P_0_, P_1_, and P_2_. (**C**) Quantitative analysis of Cx43 levels in control and ODDD patient-derived MSCs before and after 7 and 14 days of adipogenic differentiation. *, *p* < 0.05; **, *p* < 0.01 compared to undifferentiated MSCs. Data represent the standard error of the mean of three independent experiments.

**Figure 4:**
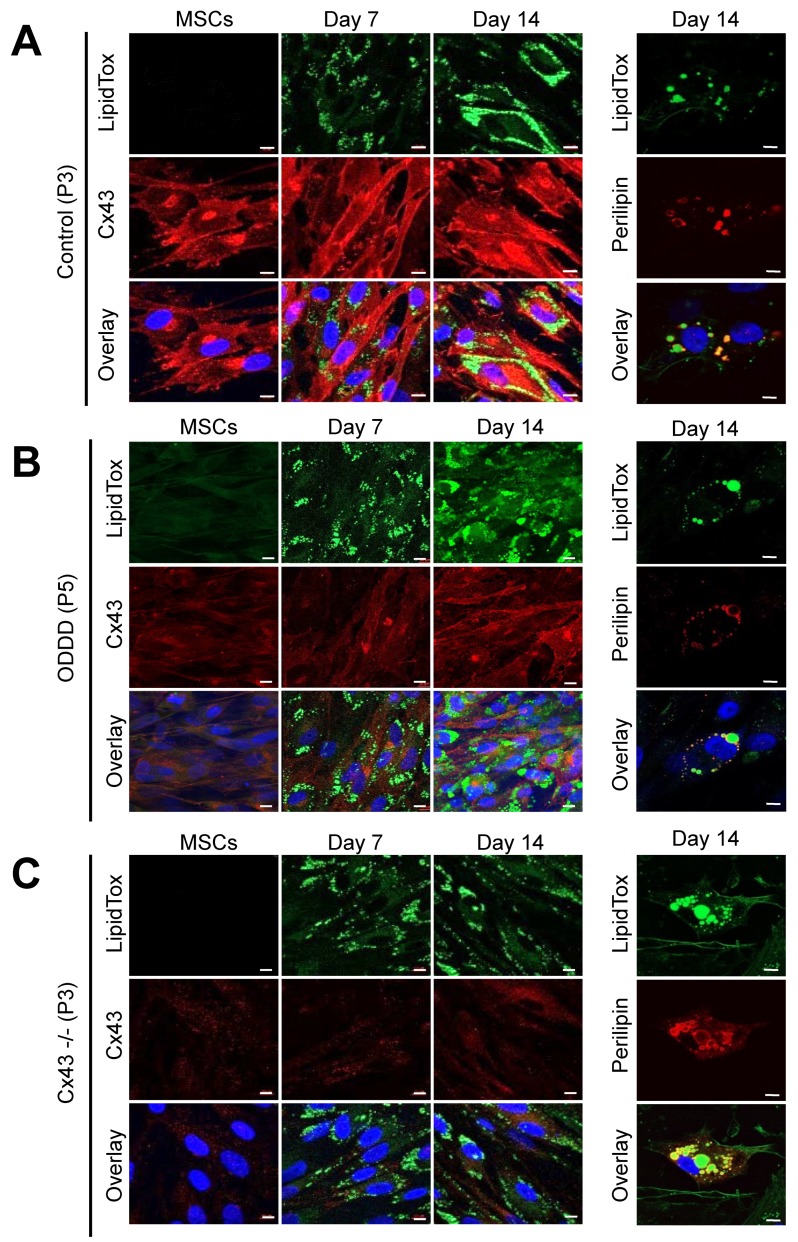
Early passage MSCs differentiate along the adipogenic lineage independent of Cx43 status. Representative images of undifferentiated (passage, P3-P5) and differentiated MSCs after staining for lipid droplets (LipidTox, green), perilipin (red), Cx43 (red), and/or Hoechst (nuclei, blue) after 0, 7, or 14 days of differentiation. (**A**) Control, (**B**) ODDD patient-derived, and (**C**) Cx43-/- MSCs were labeled at 0, 7, or 14 days after in vitro adipogenic differentiation. Scale bar = 10 μm.

To determine whether Cx43 is regulated during MSC-to-adipogenic differentiation in culture, control, ODDD patient-derived, and Cx43-/- MSCs were induced along the adipogenic pathway for 14 days in vitro (Figure 3 and Figure 4). Successful adipogenic differentiation is typically accompanied by lipid droplet accumulation within cells, which can readily be detected by LipidTox staining [41]. Indeed, both control and ODDD iPSC-derived MSCs exhibited increased lipid accumulation in multilocular vesicles after 14 days of in vitro adipogenic differentiation (Figure 3A). It was notable that ODDD-MSCs, with reduced Cx43 gap junction plaques, also successfully formed LipidTox-positive lipid droplets with lipid droplet sizes ranging from small to large, similar to that seen in control cells (Figure 3A). Interestingly, Cx43 expression significantly increased during adipogenic differentiation in both control and ODDD patient-derived MSCs at 14 days of differentiation compared to their respective undifferentiated MSCs (Figure 3B,C). Control MSCs exhibited a 1.7 ± 0.2-fold increase in Cx43 (combined P_0_, P_1_, P_2_ species) after 14 days of in vitro differentiation. In ODDD patient-derived MSCs, Cx43 levels (primarily represented by P_0_ and P_1_ species) increased by 2.1 ± 0.1-fold, as cells differentiated into lipid-positive cells (Figure 3C). Thus, while these results show that Cx43 is actively regulated during adipogenesis, the P_2_ species of Cx43 is not necessary to drive adipogenesis.

### 3.3. MSCs Expressing Cx43, a Cx43 Mutant or Lacking Cx43 Retain Adipogenic Potential

The regulated expression of Cx43 during in vitro differentiation of MSCs pointed toward a potential role for Cx43 in adipogenesis. To address this question, we induced early passage (P3–5) MSC with and without Cx43, as well as MSCs expressing an ODDD linked mutant, to differentiate along the adipogenic lineage. LipidTox green staining revealed the presence of lipid droplets after only seven days of in vitro differentiation. Lipid droplet size and frequency appeared to increase at day 14 in some MSCs regardless of their Cx43 status, but this was not remarkable, suggesting that MSCs were not actively proceeding to late stage adipogenesis (Figure 4A–C). Immunolabeling for the lipid droplet-associated protein, perilipin, revealed its presence on the surface of lipid droplets on both small and large lipid droplets after 14 days of cell differentiation (Figure 4A–C).

### 3.4. Cx43-Deficient MSCs Undergo Premature Cellular Senescence and Loss the Ability to Differentiate

**Figure 5:**
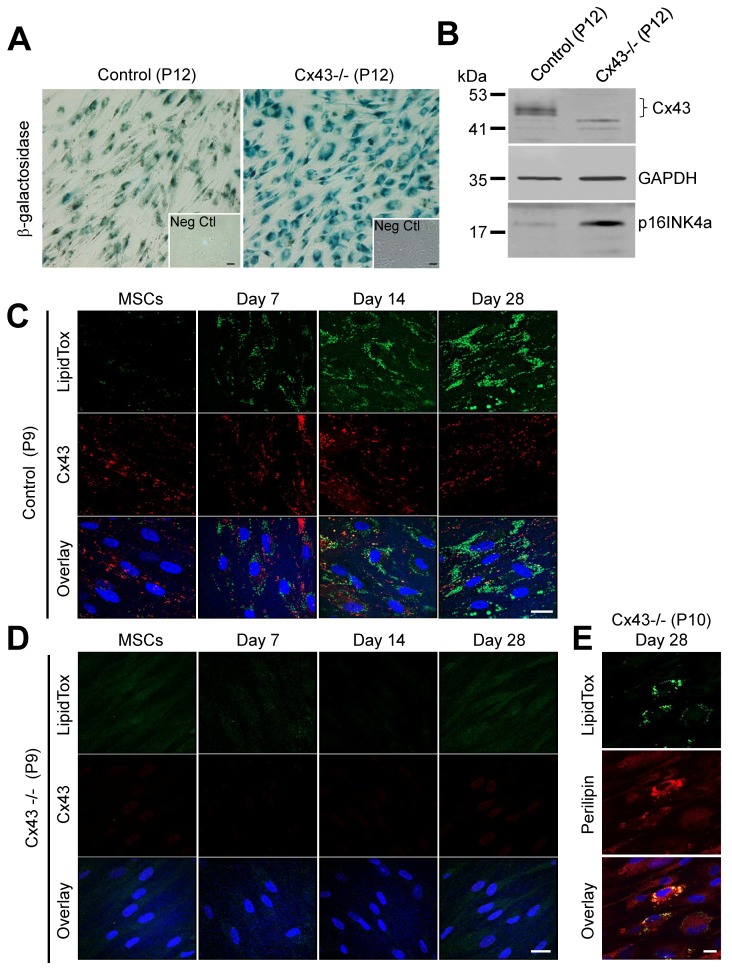
Cx43 protects MSCs from premature cellular senescence. (**A**) Representative images of senescence-associated β-galactosidase levels in control and Cx43-/- MSCs after passage 12 (P12). Inset denotes β-galactosidase staining in control MSCs passage 6. Scale bar = 50 μm (**B**) Representative immunoblot of Cx43, GAPDH, and p16INK4a in control and Cx43-/- MSCs at passage 12. (**C**,**D**) Immunofluorescent images of lipid accumulation (LipidTox, green), Cx43 (red), and nuclei (Hoechst, blue) in late passage (P9) control and Cx43-/- MSCs at 0, 7, 14, and 28 days of in vitro adipogenic differentiation. (**E**) Very few Cx43-/- MSCs at the late passage (P10) retained their adipogenic differentiation potential after 28 days of in vitro adipogenic differentiation, as noted by limited labeling for lipid markers; (LipidTox, green; perilipin, red; nuclei, blue). Scale bar = 20 μm.

We next assessed whether MSCs with various Cx43 status would maintain a similar ability to differentiate after later passage or whether a change in Cx43 status may lead to premature cell senescence and the loss of differentiation potential in late passage cells (P9–12). First, we assessed passage 9 (P9) control MSCs to reassess their CD expression profile and found that they expressed low levels of CD34 (0.2%) and CD45 (4.8%), and expressed high levels of CD73 (99.7%), CD90 (98.8%), and CD105 (99.7%), which was in agreement with what is generally expected of stem cells. However, we observed that late passage (P9-12) Cx43-/- MSCs exhibited cell morphological changes and slower growth, as noted by longer time between cell passages. Compared to control MSCs, Cx43-/- cells exhibited characteristics of cells that are beginning to senesce [42,43]. To assess whether there were population differences in the amount of senescence occurring between Cx43-positive and Cx43-negative MSCs, we first found that Cx43-negative cells expressed higher levels of β-galactosidase compared to control cells at the same passage (Figure 5A). Similarly, expression of the master regulator of cellular senescence, p16INK4a was upregulated in Cx43-/- MSCs compared to controls (Figure 5B). Strikingly, the majority of late passage (P9) Cx43-/- MSCs lost the capacity for in vitro adipogenic differentiation. However, passage-matched control MSCs retained their differentiation potential, as evidenced by the formation of LipidTox stained lipid droplets (Figure 5C,D). The inability of the bulk of the Cx43-/- cells to differentiate was evident, even when induced to differentiate up to 28 days in vitro (Figure 5D). It was notable that a select few cells that lacked Cx43 still maintained the ability to differentiate, as noted by the expression of perilipin and the positive staining with LipidTox (Figure 5E). Thus, we concluded that MSCs that continue to express Cx43 can tolerate late cell passage and retain the capacity for adipogenesis.

## 4. Discussion

MSCs are quintessential multipotent stem cells, capable of self-renewal and limited tissue-specific differentiation including differentiation into osteoblasts, chondrocytes, and adipocytes [39]. Although the gap junction protein, Cx43, has been well-established as a key regulatory of osteogenesis [10,21,22,44], less is known of the role of GJIC in adipogenesis. However, Cx43 localization and functional studies have provided considerable evidence that connexins are found in progenitor cells, leading to adipocytes and mature adipocytes from both white and brown adipose tissue [24,26,27,45,46]. In the current study, we assessed the role of Cx43 in human iPSC-derived MSCs and how the Cx43 status affects MSC differentiation along the adipogenic lineage. In short, we found that MSC differentiation along the early stages of adipogenesis was independent of Cx43. However, Cx43 was important in maintaining late-passage MSCs in a differentiation-competent state, protecting them from premature senescence. 

Since GJIC was found to be present in embryos as early at the eight-cell stage [47], it has been known that connexins play a key role in early development and in cells that have pluripotent potential. During tissue and organ development, we now know that connexins are differentially expressed, frequently leading to a population of tissue-resident stem cells that have multipotent capacity as seen with MSCs. Connexins are clearly present in many progenitor and stem cells from many sources, but how GJIC serves to guide lineage fate is only beginning to be understood. Consistent with iPSCs discoveries from other studies [48,49], we found that iPSCs-derived from dermal fibroblasts are rich in Cx43 [10], which may serve to regulate their reprogramming efficiency [49]. However, we discovered that Cx43 can be ablated from iPSCs, with no functional consequence in these cells retaining the capacity to proceed to differentiation-competent MSCs. Cx43 either functions in a hemichannel context by releasing signaling molecules to the extracellular milieu or by mediating the direct passage of signals via GJIC, both of which appear to be expendable for pluripotent iPSCs to proceed to a multipotent MSC state. This is somewhat unexpected, as there is evidence in the later stages of differentiation that Cx43 may partially govern the ability of MSCs to participate in angiogenesis and tissue repair after myocardial infarction [17,50,51,52,53,54].

When considering cells that are destined to engage in the development and maintenance of adipose tissue, both MSCs and resident adipose-derived stem cells have been shown to contain high levels of Cx43 and extensive GJIC [15,17,26]. Here, we showed that total Cx43 protein levels are highly upregulated as MSCs are induced to undergo differentiation along the adipogenic lineage. This upregulation of total protein is also seen in functionally compromised Cx43 that harbors an ODDD-linked mutation. The ODDD-linked mutation (p.V216L) examined in this study generally prevents Cx43 from acquiring its most highly phosphorylation state (P_2_), which is often associated with gap junction plaque formation and a reduction in GJIC [10]. These findings suggested that Cx43 and its upregulation may be essential to drive cell differentiation along the early stages of adipogenic lineage. However, this was not the case, as cells that had undergone complete genetic ablation of the *GJA1* (Cx43) gene continued to acquire key molecular markers (e.g., perilipin) and lipid droplets indicative of successful adipogenesis. We also observed no obvious difference in lipid droplets between Cx43-positive and Cx43-negative cell populations, as cells destined to form mature adipocytes in vivo would be expected to acquire a single lipid droplet. It is important to stress that these differentiated cells have not reached maturity with full adipocyte metabolic function that could respond to hormone signaling. Thus, our data suggest that Cx43 is expendable in at least the early stages of human MSC adipogenesis in vitro when MSCs are originally derived from Cx43-rich iPSCs. However, this study does not speak to the possible role of Cx43 in the terminal maturation of adipocytes. It was notable that when Cx43 levels increased during adipogenesis, Cx43 appeared to be retained in intracellular compartments, suggesting that there may not have been a concurrent large increase in Cx43-based GJIC. We cannot exclude the possibility that Cx43 or GJIC plays other roles in adipogenesis, as a recent study showed that GJIC inhibition by 18 α-glycerrhetinic acid or adipocyte-specific Cx43 gene knockout reduced white adipose tissue beiging and found Cx43 to be important in maintaining mitochondrial integrity in brown fat [24,25]. Another study using mouse 3T3-L1 cells reported that Cx43 phosphorylation regulated the differentiation of these precursor cells into preadipocytes, suggesting that Cx43 may play a more important in this model system [44]. Finally, we need to be cognizant that MSCs from different sources may harbor some distinct characteristics. In the case examined here, MSCs were derived from reprogrammed iPSCs, which may not fully recapitulate MSCs that are derived directly from adipose tissue or bone marrow, as recent evidence suggests that iPSC-MSCs may be less amenable to adipogenesis [55,56].

The question remains as to whether Cx43 plays an identifiable role in MSCs that might be important in the overall success of being able to differentiate these cells along the adipogenic lineage. Morphological changes and slowed cell doubling time evidence suggested that late passage cells (P9–12) lacking Cx43 were exhibiting characteristics of cells that may be entering senescence. This was confirmed as Cx43-null cells expressed high levels of β-galactosidase and p16INK4a, both indicators of cells senescing. These findings corresponded with the vast majority of cells losing the ability to differentiate, as noted by the acquisition of lipid droplets and perilipin expression. Others have demonstrated that reductions in Cx43 might serve as a biomarker of fibroblast senescence [29] and that glucose-induced downregulation of Cx43 promotes senescence in glomerular mesangial cells [57], which complement our current findings. Strikingly, Cx43 was found to prevent hematopoietic stem cell senescence by acting to transfer reactive oxygen species to bone marrow stromal cells [31]. Thus, we proposed that Cx43 is serving a critical role in maintaining the stemness properties of iPSC-induced MSCs by delaying the onset of senescence. We also found that iPSC-induced MSCs have a fragile state of stemness as attempts to use CRISPR-Cas9 to genetically ablate Cx43 directly in MSCs resulted in the loss of the expected CD molecular profile that is the hallmark of stem cells (data not shown). This finding may not be unique, as there are few reports where CRISPR-Cas9 was used to edit genes directly in cultured MSCs [1,58]. We suspect that the physical manipulations required to perform gene ablation, including transient transfection, fluorescence-activated cell sorting, and clonal selection, stressed MSCs to a point where they lose their stemness. Fortunately, iPSCs were found to be amenable to CRISPR-Cas9 Cx43 gene ablation and retained typical pluripotency signature profiles, thus providing us with a cellular platform from which to evaluate the role of Cx43 throughout each step of in vitro cell lineage specification and adipogenic differentiation. However, due to the heterogeneity of MSCs, their sensitivity to phenotypic changes during cell passage, and their inability to fully differentiate into mature adipocytes, caution needs to be exercised when assessing the molecular role of Cx43 during cell differentiation. In the future, additional studies using other human stem cell sources, such as adipose tissue-derived stem cells, should be explored to determine whether Cx43 plays a key role in their differentiation into pre-adipocytes or mature adipocytes.

## 5. Conclusions

In conclusion, we found that Cx43-mediated communication does not detectably impact human iPSC lineage commitment toward multipotent mesenchymal stem cells nor subsequent in vitro differentiation toward pre-adipocytes. However, Cx43 does delay replicative senescence of iPSC-derived MSCs in vitro, suggesting a role for cell-cell communication in the self-renewal of multipotent stem cells.

## Abbreviations

GJIC	gap junctional intercellular communication
Cx43	connexin43
iPSC	induced pluripotent stem cells
MSC	mesenchymal stem cells
ODDD	oculodentodigital dysplasia
CRISPR	Clustered Regularly Interspaced Short Palindromic Repeats
qPCR	quantitative polymerase chain reaction
